# Comparative Pharmacokinetics of Cefquinome (Cobactan 2.5%) following Repeated Intramuscular Administrations in Sheep and Goats

**DOI:** 10.1155/2014/949642

**Published:** 2014-05-19

**Authors:** Mohamed El-Hewaity, Amera Abd El Latif, Ahmed Soliman, Mohamed Aboubakr

**Affiliations:** ^1^Department of Pharmacology, Faculty of Veterinary Medicine, University of El-Sadat City, Minoufiya 32897, Egypt; ^2^Department of Pharmacology, Faculty of Veterinary Medicine, Cairo University, Giza 12211, Egypt; ^3^Department of Pharmacology, Faculty of Veterinary Medicine, Benha University, Moshtohor, Toukh, Qalyubia 13736, Egypt

## Abstract

The comparative pharmacokinetic profile of cefquinome was studied in sheep and goats following repeated intramuscular (IM) administrations of 2 mg/kg body weight. Cefquinome concentrations in serum were determined by microbiological assay technique using *Micrococcus luteus* (ATCC 9341) as test organism. Following intramuscular injection of cefquinome in sheep and goats, the disposition curves were best described by two-compartment open model in both sheep and goats. The pharmacokinetics of cefquinome did not differ significantly between sheep and goats; similar intramuscular dose rate of cefquinome should therefore be applicable to both species. On comparing the data of serum levels of repeated intramuscular injections with first intramuscular injection, it was revealed that repeated intramuscular injections of cefquinome have cumulative effect in both species sheep and goats. The *in vitro* serum protein-binding tendency was 15.65% in sheep and 14.42% in goats. The serum concentrations of cefquinome along 24 h after injection in this study were exceeding the MICs of different susceptible microorganisms responsible for serious disease problems. These findings indicate successful use of cefquinome in sheep and goats.

## 1. Introduction

Cephalosporins are described as *β*-lactam antibiotics, based on their common structural feature, containing the *β*-lactam ring. A major advantage of the *β*-lactam antibiotics is high degree of safety in the target animal [1]. Cefquinome, an aminothiazolyl cephalosporin, is a member of the fourth-generation of cephalosporins that has been used for veterinary use only [2]. It has broad-spectrum antibacterial activity against clinically important bacteria such as* streptococcus spp*,* staphylococcus spp*,* pseudomonas spp*,* E. coli,* and gram-positive anaerobes [3, 4]. It has been approved for the treatment of respiratory diseases, acute mastitis, and foot rot in cattle [5, 6]. The objective of the current study is to determine whether there are differences between sheep and goat in the disposition of cefquinome following repeated intramuscular administrations of 2 mg/kg b.wt. once daily for three consecutive days in sheep and goat, to determine if the drug has a cumulative effect after repeated intramuscular administrations and to recommend appropriate dose regimen for cefquinome in sheep and goat.

## 2. Materials and Methods

### 2.1. Antimicrobial Agent

Cefquinome was obtained from Intervet International Company, Cairo, Egypt, under a trade name: Cobactan 2.5%.

### 2.2. Animals

Five healthy nonlactating female Egyptian Baladi sheep (weighing 29–37 kg b.wt.) and five healthy nonlactating female Egyptian Baladi goats (weighing 22–28 kg b.wt.) were used. Animals were housed in hygienic stable, fed on barseem, Drawa and Concentrate. Water was provided* ad-libitum*. None of the animals were treated with antibiotics for one month prior to the trial. The experiment was performed in accordance with the guidelines set by the Ethical Committee of El-Sadat city University, Egypt.

#### 2.2.1. Experimental Design

Each animal of sheep and goats was injected intramuscularly with 2 mg/kg b.wt. cefquinome (Cobactan 2.5%) into the deep gluteal muscle of hindquarter [7] once daily for three consecutive days. Following repeated intramuscular injections in sheep and goats, three milliliters of blood were collected from the jugular vein at 5, 15, and 30 minutes, 1, 2, 4, 8, 12, and 24 h after each injection. Blood samples were left to clot for 1 hour at room temperature; the clear sera were separated by centrifugation at 3000 r.p.m for 15 minutes and stored at −20°C until assayed.

#### 2.2.2. Drug Bioassay

Concentrations of cefquinome in serum samples were determined by the microbiological assay method described by Arret et al. [8] using* Micrococcus luteus* (ATCC 9341) as test organism [9]. This method estimated the level of drug having antibacterial activity, without differentiating between the parent drug and its active metabolites. The application of microbiological assay for measuring cefquinome concentration is suitable [9]. Six wells were made at equal distances in standard Petri dishes containing 25 mL seeded agar. The wells were filled with 100 *μ*L of either the test samples or the cefquinome standard concentrations. The plates were kept at room temperature for 2 h before being incubated at 37°C for 18 h. Zones of inhibition were measured using micrometers, and the cefquinome concentrations in the test samples were calculated from the standard curve. Cefquinome (Cobactan 2.5%) standard solution of concentrations from 0.098 to 25 *μ*g/mL was prepared in antibiotic-free sheep and goat serum and phosphate buffer saline. Standard curves of cefquinome were prepared in antibacterial-free goat serum by the appropriate serial dilution. The standard curve in sheep and goat serum was linear over the range from 0.098 to 25 *μ*g/mL and the value of correlation coefficient (*r*) was 0.991. The limit of quantification was 0.098 *μ*g/mL. Protein binding of cefquinome (Cobactan 2.5%) was estimated according to Craig and Suh [10].

### 2.3. Pharmacokinetic Analysis

A pharmacokinetic computer program (R-strip, Micro-math, Scientific software, USA) was used to determine the least squares best-fit curve for cefquinome concentration versus time data. Following I.M administrations, the appropriate pharmacokinetic model was determined by visual examination of individual concentration-time curves and by application of Akaike*ʼ*s information criterion (AIC) [11]. This program also calculated noncompartmental parameters using the statistical moment theory [12]. The pharmacokinetic parameters were reported as mean ± SE. Mean pharmacokinetic parameters after repeated IM administrations were statistically compared in sheep and goats using Student's* t*-test [13].

## 3. Results

**Figure 1:**
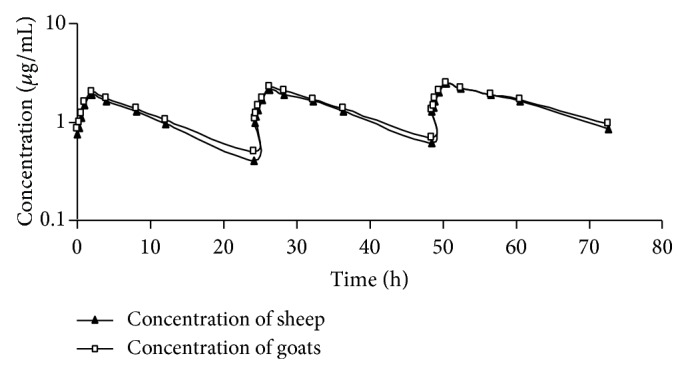
Semilogarithmic graph depicting the time-concentration of cefquinome in serum of sheep and goats after repeated intramuscular injections of 2 mg/kg b.wt. once daily for three consecutive days.

No clinical signs of adverse effects or intolerance were observed to cefquinome IM injection in sheep and goats. The mean serum concentrations of cefquinome in sheep and goat receiving repeated IM injections of 2 mg/kg b.wt. once daily for three consecutive days versus time are summarized in Figure 1. These data are best fitted to a two-compartment open model. The results illustrated nonsignificant increase in the serum level of cefquinome in goats compared to values recorded in sheep. Also the results showed a significant increase in serum concentrations of cefquinome after repeated doses compared to the first dose in both species sheep and goat. Cefquinome could be detected in a therapeutic concentration for 24 h post IM injection in sheep and goats. The pharmacokinetic parameters of cefquinome following repeated IM injections of 2 mg/kg b.wt. once daily for three consecutive days in sheep and goats are presented in Table 1. There were no significant differences between the pharmacokinetic parameters of cefquinome in sheep and goats after repeated IM doses. The result of* in vitro* protein binding study indicated that 15.65% and 14.42% of cefquinome were bound to sheep and goats serum protein, respectively.

## 4. Discussion

The disposition of cefquinome following intramuscular administration in sheep and goat was best described by a two-compartment open model which was similar to that described in sheep [7, 14], piglets [15], and ducks [16]. However, a monocompartment open model was shown to provide the best fit for intramuscular cefquinome plasma concentration-time data in goat [17] and camels [18].

Following first intramuscular injection of cefquinome, the mean peak serum concentrations (*C*_max⁡_) were 1.80 ± 0.09 and 1.88 ± 0.10 *μ*g/mL in sheep and goats, respectively. These concentrations were achieved at times (*T*_max⁡_) 2.61 ± 0.11 and 2.62 ± 0.09 h in sheep and goats, respectively. These results indicate the slow absorption of this formula. These results differ from those recorded in sheep (*C*_max⁡_) 2.60 ± 0.14 *μ*g/mL at (*T*_max⁡_) 0.50 h [7] and goats (*C*_max⁡_) 4.84 ± 0.23 *μ*g/mL at (*T*_max⁡_) 1.50 h [17]. Such differences are common and frequently related to interspecies variation, assay methods used, age, breed and health status of the animal, and the formulation of the drug used [19].

The absorption half-life of cefquinome following intramuscular injection in sheep and goats was 0.76 ± 0.036 h and 0.73 ± 0.016 h which was similar to the *T*_0.5ab_ of 0.664 h reported in one-year-old sheep [14] and to the *T*_0.5ab_ of 0.64 h reported in goats [17]. However, a shorter absorption half-life of cefquinome has been reported in ducks (0.12 h) and chicken (0.17 h) after intramuscular injection [16–20] indicating longer duration for the drug to reach systemic circulation and slower onset of pharmacological action in sheep and goats.

Cefquinome showed long elimination half-life (*T*_0.5el_) after intramuscular injection in sheep and goats 9.03 ± 0.89 h and 10.14 ± 1.42 h, respectively; prolonged elimination half-life has been reported for cefquinome in buffalo calves, cattle calves, cows, and goats 12.86, 13.46, 7.10, and 8.68 h, respectively [21]. However, a shorter elimination half-life has been reported in sheep (2.41 h) and goats (5.86 h) after intramuscular injection [14–17]. Such differences are common and frequently related to interspecies variation, assay methods used, and the formulation of the drug used [19].

The mean residence time (MRT) of cefquinome was 14.23 ± 1.10 h in sheep and 15.16 ± 1.44 h in goats which was consistent with value recorded in camels 16.74 h [18]. The longer *T*_0.5el_ and MRT of cefquinome in the present study indicated long persistence of the drug.

There were no significant differences between the pharmacokinetic parameters of cefquinome in sheep and goats after repeated intramuscular doses. The results were similar to data recorded by Craigmill et al. [22] who found that no significant differences between the pharmacokinetic parameters following intravenous administration of amoxicillin in sheep and goats. Also the results were consistent with those reported by Elsheikh et al. [23] who found that the pharmacokinetics of enrofloxacin did not differ significantly between sheep and goats following intravenous and intramuscular administration.

The relative higher serum concentrations of cefquinome after repeated doses compared to the first dose indicated the accumulation of cefquinome in blood during multiple doses at 24 hours intervals for three consecutive days in sheep and goats. These observations agreed with data reported by El-Banna and Abo El-Sooud [24] who found that progressive daily increase in the mean serum concentrations following the repeated intramuscular injection of ciprofloxacin in lactating goats in a daily dose of 5 mg/kg b.wt. for five consecutive days.

*In vitro* protein binding percent of cefquinome in sheep and goat serum was 15.65% and 14.42%, respectively, so it could be considered as slightly serum protein binding [25].These results were similar to those recorded in sheep 13.002% [14].

The* in vitro* efficacy of cefquinome against a wide range of Gram-negative and Gram-positive bacterial pathogens has been demonstrated by various workers. Considering the reported MIC_90s_ (0.06–0.39 *μ*g/mL) for* Escherichia coli*,* Pasteurella multocida,* and* Streptococcus agalactiae* [26–31]. In this discussion an average MIC_90_ of 0.125 *μ*g/mL of cefquinome has been considered. Based on this data, the intramuscular injection of cefquinome at a dose of 2 mg/kg at 24 h interval is sufficient to maintain serum concentration above MIC_90_ for most sensitive susceptible pathogens (0.125 *μ*g/mL); these findings indicate the suitability of successful use of cefquinome in sheep and goats. A recommended single daily dose of 2 mg/kg of cefquinome given intramuscularly achieves therapeutic concentrations in serum exceeding the MIC_90s_ against different susceptible pathogens in sheep and goats.

## 5. Conclusion

Based on this study, there were no significant differences between the pharmacokinetic parameters of cefquinome (Cobactan 2.5%) in sheep and goats after repeated intramuscular doses, so that an optimal intramuscular dosage regimen of cefquinome (Cobactan 2.5%) would be 2 mg/kg body weight once daily in sheep and goats to achieve and maintain the therapeutic serum levels within a safe limit.

## Figures and Tables

**Table 1:** Mean (±SE) kinetic parameters of cefquinome following repeated intramuscular injections of 2 mg/kg b.wt. once daily for three consecutive days in sheep and goats.

Parameter	Unit	Sheep			Goat		
		1st day	2nd day	3rd day	1st day	2nd day	3rd day
*A *	*µ*g·mL^−1^	1.75 ± 0.10	1.72 ± 0.06	1.70 ± 0.12	1.73 ± 0.08	1.75 ± 0.09	1.56 ± 0.13
*K*_ab_	h^−1^	0.91 ± 0.036	0.85 ± 0.06	0.97 ± 0.02	0.93 ± 0.015	0.83 ± 0.035	1.03 ± 0.02
*T*_0.5(ab)_	h	0.76 ± 0.036	0.82 ± 0.02	0.73 ± 0.02	0.73 ± 0.016	0.82 ± 0.03	0.67 ± 0.02
*B*	*µ*g·mL^−1^	2.37 ± 0.14	2.58 ± 0.10	2.82 ± 0.21	2.43 ± 0.09	2.71 ± 0.08	2.74 ± 0.13
*K*_el_	h^−1^	0.074 ± 0.002	0.057 ± 0.004	0.05 ± 0.005	0.068 ± 0.003	0.057 ± 0.002	0.044 ± 0.002
*T*_0.5(el)_	h	9.03 ± 0.89	11.35 ± 1.40	14.01 ± 0.99	10.14 ± 1.42	11.57 ± 1.28	15.71 ± 1.52
*C*_max⁡_	*µ*g·mL^−1^	1.80 ± 0.09	2.03 ± 0.14	2.30 ± 0.10	1.88 ± 0.10	2.15 ± 0.09	2.38 ± 0.08
*T*_max⁡_	h	2.61 ± 0.11	2.77 ± 0.21	2.70 ± 0.15	2.62 ± 0.09	2.88 ± 0.19	2.62 ± 0.13
AUC_(0-inf)_	*µ*g·h·mL^−1^	29.96 ± 1.20	40.61 ± 3.16	54.98 ± 4.21	31.11 ± 1.05	45.22 ± 2.08	61.20 ± 3.44
MRT	h	14.23 ± 1.10	16.98 ± 1.75	20.60 ± 1.31	15.16 ± 1.44	16.97 ± 0.88	23.06 ± 2.78
IBD	h	27.91 ± 3.53	—	—	28.82 ± 4.88	—	—

*A*: zero-time intercept of distribution phase; *K*_ab_: first-order absorption rate constant; *T*_0.5(ab)_": absorption half-life; *B*: zero-time intercept of elimination phase; *K*_el_: first-order elimination rate constant; *T*_0.5(el)_: elimination half-life; *C*_max⁡_: maximum serum concentration; *T*_max⁡_: time to peak serum concentration; AUC_(0-inf)_: area under serum concentration-time curve; MRT: mean residence time; IBD: interval between doses.
